# Altered Homotopic Functional Connectivity Within White Matter in the Early Stages of Alzheimer’s Disease

**DOI:** 10.3389/fnins.2021.697493

**Published:** 2021-09-22

**Authors:** Pan Wang, Zedong Wang, Jianlin Wang, Yuan Jiang, Hong Zhang, Hongyi Li, Bharat B. Biswal

**Affiliations:** ^1^Ministry of Education (MOE) Key Laboratory for Neuroinformation, Center for Information in Medicine, School of Life Sciences and Technology, The Clinical Hospital of Chengdu Brain Science Institute, University of Electronic Science and Technology of China, Chengdu, China; ^2^The Fourth People’s Hospital of Chengdu, Chengdu, China; ^3^Department of Biomedical Engineering, New Jersey Institute of Technology, Newark, NJ, United States

**Keywords:** Alzheimer’s disease, DTI, homotopic functional connectivity, mild cognitive impairment, support vector machine

## Abstract

Alzheimer’s disease (AD) is a progressive neurodegenerative disorder with memory loss and cognitive impairment. The white matter (WM) BOLD signal has recently been shown to provide an important role in understanding the intrinsic cerebral activity. Although the altered homotopic functional connectivity within gray matter (GM-HFC) has been examined in AD, the abnormal HFC to WM remains unknown. The present study sought to identify changes in the WM-HFC and anatomic characteristics by combining functional magnetic resonance imaging with diffusion tensor imaging (DTI). Resting-state and DTI magnetic resonance images were collected from the OASIS-3 dataset and consisted of 53 mild cognitive impairment (MCI) patients, 90 very MCI (VMCI), and 100 normal cognitive (NC) subjects. Voxel-mirrored HFC was adopted to examine whether WM-HFC was disrupted in VMCI and MCI participants. Moreover, the DTI technique was used to investigate whether specific alterations of WM-HFC were associated with anatomic characteristics. Support vector machine analyses were used to identify the MCI and VMCI participants using the abnormal WM-HFC as the features. Compared with NC, MCI, and VMCI participants showed significantly decreased GM-HFC in the middle occipital gyrus and inferior parietal gyrus and decreased WM-HFC in the bilateral middle occipital and parietal lobe-WM. In addition, specific WM-functional network alteration for the bilateral sub-lobar-WM was found in MCI subjects. MCI subjects showed abnormal anatomic characteristics for bilateral sub-lobar and parietal lobe-WM. Results of GM-HFC mainly showed common neuroimaging features for VMCI and MCI subjects, whereas analysis of WM-HFC showed specific clinical neuromarkers and effectively compensated for the lack of GM-HFC to distinguish NC, VMCI, and MCI subjects.

## Introduction

Alzheimer’s disease (AD) is a progressive neurodegenerative disease characterized clinically by memory loss and cognitive impairment. Both mild cognitive impairment (MCI) and very MCI (VMCI) are two crucial transitional states between normal aging and neurodegenerative AD, but VMCI is generally considered a precursor of MCI (Petersen et al., 1999; Storandt et al., 2006). Accumulating brain imaging studies have demonstrated abnormal morphological and functional magnetic resonance imaging (fMRI) in MCI and AD subjects (Kantarci et al., 2001; Mito et al., 2018; Nho et al., 2019). However, few studies have compared the pathological mechanism of neuroimaging between VMCI and MCI. Moreover, a comprehensive characterization of the standard features and specific alterations between VMCI and MCI subjects has important applications in the treatment and prognosis of AD.

Because resting-state homotopic functional connectivity (HFC) was a key characteristic of the intrinsic functional architecture in the human brain (Salvador et al., 2008; Stark et al., 2008). Zuo et al. (2010) developed a voxel-mirrored homotopic connectivity method (VMHC) to investigate the functional connectivity between each voxel and its mirrored counterpart in hemispheres. Subsequently, this method was used to investigate the aberrant interhemispheric functional connectivity in various disorders, such as autism (Anderson et al., 2011), epilepsy (Ji et al., 2014; Liu et al., 2016), schizophrenia (Liao et al., 2019), cocaine addiction (Kelly et al., 2011), and AD (Wang et al., 2015). However, these studies have primarily focused on the brain’s gray matter (GM). The signals from white matter (WM) have usually been considered as noises and are rarely reported in the literature.

The WM densely connects different regions of GM and accounts for nearly half of the human brain (Teo et al., 1997; Zhang and Sejnowski, 2000; Harris and Attwell, 2012). The WM BOLD signal has recently been shown to provide an important role in understanding the intrinsic cerebral activity (Ji et al., 2017; Peer et al., 2017; Ding et al., 2018; Li et al., 2019; Wang et al., 2020, 2021). Ji et al. (2017), in a seminal study, first demonstrated that the BOLD signal in WM varied with physiological states and was correlated with structural features. Ding et al. (2018) have found that BOLD signals in certain WM tracts are functionally correlated with specific GM regions during different tasks. Peer et al. (2017) first reported the existence of symmetric WM functional networks (WM-FNs) closely related to GM networks and the underlying structural WM tracts. In our previous study, we have demonstrated the reproducible WM-FNs and that the corpus callosum had a unique spatial distribution pattern corresponding to different WM-FNs (Wang et al., 2020, 2021). As the WM structural alterations have characterized many brain disorders, researchers have further explored the functional anomaly within WM during rest, such as schizophrenia (Jiang et al., 2019), Parkinson’s disease (Ji et al., 2019), and pontine strokes (Wang et al., 2019). However, few studies have investigated the abnormal homotopic functional connectivity within WM (WM-HFC) in patients within the brain (Zhao et al., 2019; Gao et al., 2020).

Diffusion tensor imaging (DTI) examines the microstructural properties of the WM and provides important information on *in vivo* neuronal fiber tracts (Chua et al., 2008). Tractography, a method to extract neuronal fiber tract information using the diffusion tensor MRI, can assess AD progression. For instance, WM tracts in the corpus callosum have revealed significant changes as patients progressed from NC to MCI and finally to AD (Douaud et al., 2011). As DTI provides unique information on WM and three-dimensional visualization of a neuronal pathway, the current study explored the abnormal WM-HFC and the anatomical characteristics of abnormal WM-HFC regions by combing resting-state fMRI and DTI.

We hypothesize abnormal interhemispheric connectivity in VMCI and MCI subjects compared with normal cognitive (NC) subjects. To this end, VMHC was adopted to examine whether WM-HFC was disrupted in VMCI and MCI subjects. Moreover, the DTI technique was used to investigate whether specific regional functional changes within WM were associated with the disrupted anatomical characteristic. Finally, the support vector machine (SVM) analysis was performed to identify the VMCI/MCI using the above abnormal WM-HFC as features.

## Materials and Methods

This study had used publicly available data from the OASIS-3 dataset^1^ and consisted of 53 MCI subjects, 90 VMCI subjects, and 100 NC subjects. These data were collected across several ongoing studies in the Washington University Knight Alzheimer’s Disease Research Center. The dementia status of all patients was assessed using the Clinical Dementia Rating (CDR) Scale (Morris, 1993), with CDR = 0 (NC), CDR = 0.5 (VMCI), and CDR = 1 (MCI). The severity of cognitive impairment was assessed based on the Mini-Mental State Examination (Folstein et al., 1975). Written informed consent was obtained from all participants before fMRI or neurologic evaluations. Additionally, clinical scale information was obtained from all the patients’ subjects. The detailed information is shown in Table 1.

**TABLE 1:** Demographics and clinical characteristics of subjects.

Characteristics	NC (*N* = 100)	VMCI (*N* = 90)	MCI (*N* = 53)	*P*-value
Age	74.41 ± 8.227	74.76 ± 7.668	75.89 ± 8.554	*P* = 0.494^a^
Sex (M/F)	50/50	48/42	33/20	*P* = 0.347^b^
Education	14.32 ± 1.757	14.59 ± 3.016	14.75 ± 3.204	*P* = 0.844^a^
Mean FD	0.282 ± 0.150	0.302 ± 0.176	0.315 ± 0.149	*P* = 0.281^a^
MMSE	28.86 ± 1.318	25.99 ± 2.882	22.08 ± 4.057	*P* < 0.001^a^
Handness (L/R)	0/100	0/90	0/53	

*M, male; F, female; MMSE, Mini-Mental State Examination; Mean FD, mean framewise displacement.*

*^*a*^One-way ANOVA (using non-parametric test).*

*^*b*^Chi-square.*

We obtained the diffusion data from 26 MCI, 44 VMCI, and 43 NC from the functional analysis dataset discussed earlier in the study. These subjects of diffusion data have matched the age, sex, and education. The detailed clinical information is shown in Supplementary Table 1. Because the current study focused on classifying MCI, VMCI, and NC based on the SVM analysis, we also obtained the subjects as testing data collected using another fMRI machine with 3-T Siemens’s Biograph scanners (8 MCI, 18 VMCI, and 14 NC).

### Data Acquisition

All patients and healthy controls were collected with 3-T Siemens’s Trio Tim scanners. They were instructed to rest with their eyes closed, lie still, not to think anything in particular, and not to fall asleep during scanning. Resting-state functional images were collected using the following parameters: voxel size = 4 × 4 × 4 mm^3^, echo time = 27 ms, repetition time = 2.2 s, flip angle = 90°, slice thickness = 4 mm, and number of time points = 164. The high-spatial-resolution three-dimensional T1-weighted anatomic images were acquired using following parameters: voxel size = 1 × 1 × 1 mm^3^, echo time = 3.16 ms, repetition time = 2.4 s, flip angle = 8°, slice thickness = 1 mm. DTI covering the whole brain was obtained using an echo-planar imaging sequence, including 24 volumes with diffusion gradients applied along 24 non-collinear directions. The parameters of DTI were as follow: voxel size = 2 × 2 × 2 mm^3^, echo time = 0.112 s, repetition time = 14.5 s, flip angle = 90°, slice thickness = 2 mm.

### Data Preprocessing

#### Functional Images

fMRIs were preprocessed using the Data Processing Assistant for Resting-State fMRI^2^ and SPM12 software^3^ toolkits. The preprocessing steps included: excluding the first five volumes from each fMRI data to allow for T1 relaxation stabilization, correcting for head motion-related signal changes. Individual T1-weighted structural images were co-registered to the mean functional image. Each structural image was segmented into GM, WM, and cerebrospinal fluid (CSF) using the DARTEL segmentation algorithm. Linear trends were removed to correct for signal drift. The mean signals from CSF and 24 rigid body motion parameters (6 head motion parameters, 6 values at previous time points of 6 head motion parameters, and the 12 corresponding squared items) were regressed from the functional images. Scrubbing using motion “spikes” was performed as separate regressors identified by framewise displacement greater than 1 mm to further reduce the effect of head motion. The head motion scrubbing regressors were used in this study, as it is effective in eliminating the effect of head motion at the spike on the signal without changing the correlation values (Power et al., 2012; Satterthwaite et al., 2013). Temporal filtering was done in the low-frequency range of 0.01–0.1 Hz. To avoid mixing WM and GM signals, the functional images were minimally spatially smoothed (4 mm full-width half-maximum, isotropic) separately within WM and GM templates for each subject. The WM and GM voxels were identified using the segmentation results from each subject (using a threshold of 0.5 using SPM12’s tissue segmentation). After smoothing, the functional image was normalized into MNI space in 3 × 3 × 3 mm^3^.

To account for differences in the geometric configuration of the cerebral hemispheres, we further transformed the preprocessed functional images to a symmetric space using the following procedures: (a) the normalized T1 images were averaged across all the subjects to create a mean T1 image template; (b) create a symmetric T1 template by averaging the mean T1 template with its left–right mirrored version; (c) normalized T1 images were registered to the symmetric template and applied to the non-linear transformation to the normalized functional images. Finally, we calculated the Pearson correlation coefficient between the time series of every pair of symmetrical interhemispheric voxels. The resulting correlations for each paired voxel constituted a VMHC map. These VMHC maps were standardized by Fisher’s z transform so that individual maps could be averaged and compared among participants.

#### Diffusion Tensor Images

Diffusion tensor images were preprocessed and analyzed using FSL^4^ and DSI Studio.^5^ For each subject, the EDDY tool was used to correct eddy current distortions and subject movements (Yamada et al., 2014). For each subject, the axial diffusivity (AD), fractional anisotropy (FA), and mean diffusivity (MD) were computed based on the voxel-wise estimates and then saved in the DSI studio.

Several regions showing abnormal WM-HFC in patients were selected as regions of interest (ROIs) for subsequent analysis of DTI data. Before we obtained the DTI indexes, the space for abnormal WM-HFC regions was transformed from MNI space to individual default diffusion space of DSI studio.

### Creation of Group Functional Gray Matter/White Matter Symmetric Mask

Each structural image was segmented to identify each voxel in the whole brain as belonging to the GM, WM, or CSF. The group GM symmetric mask was produced using the following procedures: (a) the normalized GM probability maps were averaged across all the subjects to obtain a mean group GM mask; (b) a symmetric group GM mask was created by averaging the mean group GM mask with its left–right mirrored version. Finally, a binarized group GM symmetric mask was obtained by thresholding symmetric group GM mask defined earlier at 0.2 in line with previous WM functional study to obtain the group GM mask using the same threshold (Peer et al., 2017). Using the same method, a binarized group WM symmetric mask was also obtained by thresholding the symmetric group WM mask defined earlier at 0.8. In addition, as the SPM segment did not exclude the subcortical nucleus from the WM, we also obtained the group WM symmetric mask excluding the subcortical nucleus from the WM referring to previous WM functional study (Ji et al., 2019). Based on the group WM mask mentioned earlier, we have further estimated the effect on our results after excluding the subcortical nucleus from the WM mask (Supplementary Material 3).

### Statistical Analysis

The one-way analysis of variance (ANOVA) was performed using the DPABI software (DPABI_V6.0_210501),^6^ and *post hoc* analysis was performed to calculate the two-sample *t*-test using the GraphPad software.^7^ Abnormal regions of HFC within-group GM symmetric mask among three groups were identified using one-way ANOVA, with age, sex, and education as covariates [*p* < 0.05, false discovery rate (FDR)-corrected and one-tailed]. Two pairs of bilateral ROIs were obtained for *post hoc* analysis and were compared using the two-sample *t*-test with age, sex, and education as covariates [two-tailed, *p* < 0.05, Bonferroni-corrected for multiple comparisons (*p* < 0.05/6)].

The one-sample *t*-test was calculated for the individual WM-HFC maps across participants in each of the three samples within the group WM symmetric mask (*p* < 0.05, FDR-corrected). Using a similar statistical analysis, the abnormal regions of WM-HFC were obtained among three groups using one-way ANOVA (*p* < 0.05, FDR-corrected and one-tailed). Three pairs of bilateral ROIs were obtained for *post hoc* analysis and were compared using the two-sample *t*-test, with age, sex, and education as covariates (two-tailed, Bonferroni-corrected for multiple comparisons, *p* < 0.05/9). For each pair of bilateral ROIs within WM, the bilateral anatomic characteristics within WM were further analyzed among three groups. Mean AD, FA, and MD in the ROIs of abnormal WM-HFC were compared between three groups using a two-sample *t*-test (Bonferroni-corrected for multiple comparisons, *p* < 0.05/9).

### Classification Model Based on Homotopic Functional Connectivity Within White Matter

To investigate the classification at early stages of AD, SVM was performed to identify patient participants from healthy controls and discriminate MCI and VMCI subjects using the LIBSVM software package (Chang and Lin, 2011). The previous study has demonstrated that the SVM was a useful classification approach that helped identify the patients at the individual level (Auria and Moro, 2008). SVM analysis was performed using 166 voxel signals of each subject in the three ROIs obtained from the WM-HFC analysis. SVM classification diagnoses were performed using the voxel signals, age, sex, education, and Mini-Mental State Examination information in line with previous SVM analysis (Matsuo et al., 2019). The kernel function for SVM analysis was selected to be linear. In addition, we have used the optimization of hyper-parameters to estimate the c parameter (cmin, cmax, cstep = 2^–8^, 2^3^, 2^–2^). To improve the accuracy of multiclass SVM (NC vs. VMCI vs. MCI), we performed feature optimization. Specifically, for each feature, we have performed three two-sample *t*-tests between NC, VMCI, and MCI (NC vs. VMCI, NC vs. MCI, and VMCI vs. MCI). If all three *p*-values were less than 0.05, we kept this feature; otherwise, the feature was excluded from the total features. All features were scaled to have unit variance. Consequently, we obtained one classification model for multiclass SVM and then directly applied it to the validation samples. The validation samples were collected using a different 3T Siemens MRI scanner.

## Results

### Homotopic Functional Connectivity Within Gray Matter

**FIGURE 1:**
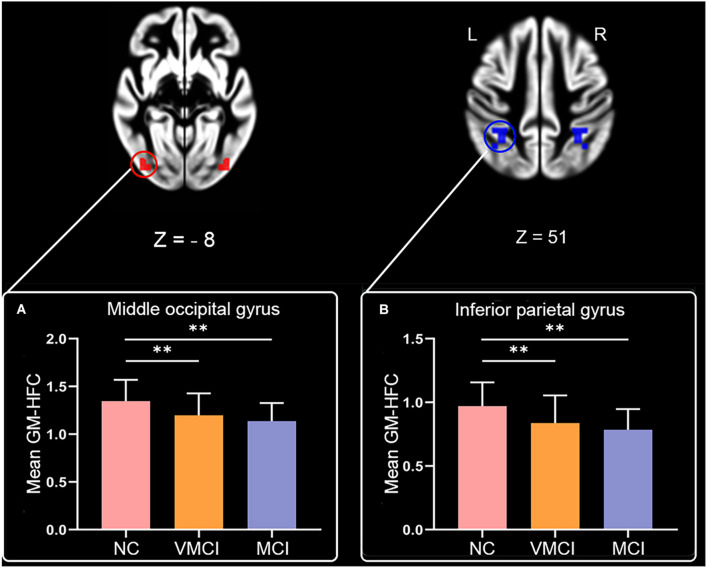
Abnorma homotopic functional connectivity within GM among groups. Magnetic resonance images maps show abnormal GM-HFC among groups (*P* < 0.05, FDR-corrected). Histogram shows results of *post hoc* analysis between three groups. **(A,B)** The mean GM-HFC for the middle occipital gyrus and inferior parietal gyrus, respectively. Statistical significance level was set at *P* < 0.05. ^∗∗^denotes Bonferroni correction with *p* < 0.05/6.

The current study reported the results using the viewer function in the DPABI package (see text footnote 6). We have described the location of result regions using the Automated Anatomical Labeling atlas containing 116 regions (90 cortical/subcortical and 26 cerebellar/vermis regions) (Tzourio-Mazoyer et al., 2002). Within-group analysis for homotopic functional connectivity within GM (GM-HFC) indicated that NC, VMCI, and MCI subjects (Supplementary Figures 1A–C) had robust regional differences (*p* < 0.05, FDR-corrected). We found that GM-HFC maps for the NC group exhibited the strongest connection, followed by the VMCI group (GM-HFC_N__C_ > GM-HFC_VMCI_ > GM-HFC_MCI_) (Supplementary Figure 1), which was similar to the previous amplitude of low-frequency fluctuation (ALFF) study pattern (ALFF_N__C_ > ALFF_VMCI_ > ALFF_MCI_) (Desikan et al., 2006). The ALFF is a resting-state fMRI parameter that measures the regional intensity of spontaneous fluctuations in the BOLD signal and reflects the neural activity of a specific brain region (Zang et al., 2012). Significantly abnormal GM-HFC among three groups is shown in Figure 1 and Table 1. Specifically, GM-HFCs for the bilateral middle occipital gyrus and inferior parietal gyrus were lower in MCI and VMCI subjects than in NC subjects with GM-HFC_N__C_ > GM-HFC_VMCI_ > GM-HFC_MCI_.

### Homotopic Functional Connectivity Within White Matter

**FIGURE 2:**
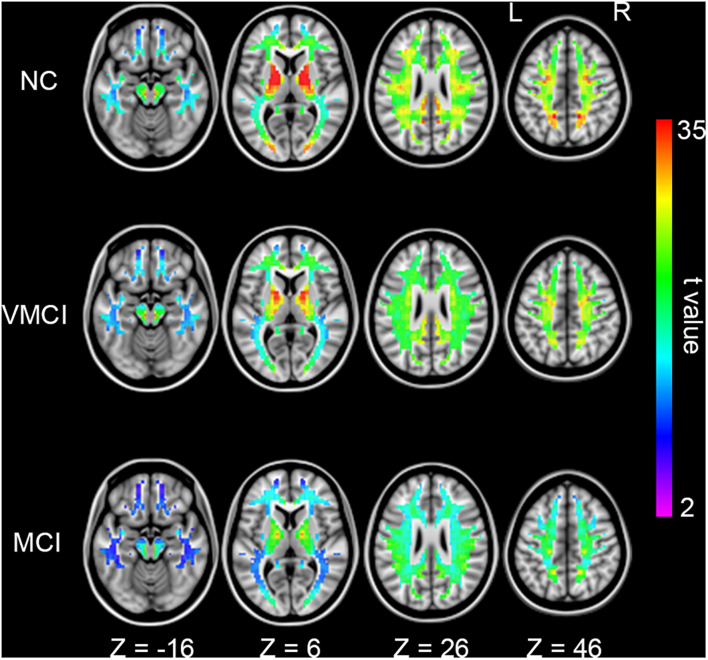
Homotopic functional connectivity within WM for each group. Colored voxels indicate significant functional connectivity with voxels at symmetric positions of other hemispheres (*p* < 0.05, FDR corrected).

**FIGURE 3:**
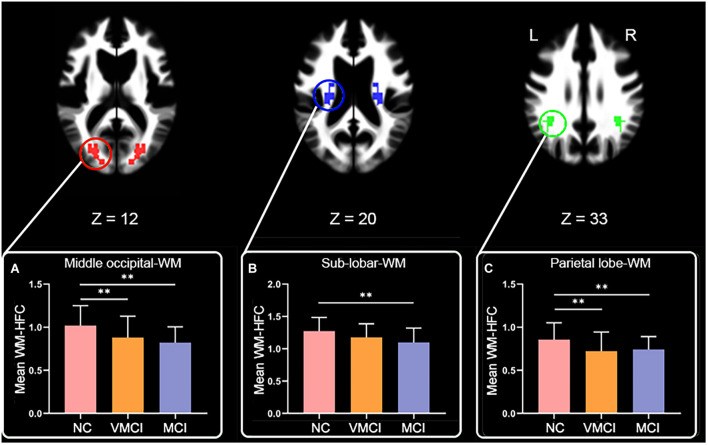
Abnormal homotopic functional connectivity within WM among groups. Brain slices and histogram, respectively, show abnormal regions and results of *post hoc* analysis. **(A–C)** The mean WM-HFC for the middle occipital-WM, sub-lobar-WM, and parietal lobe-WM, respectively. ^∗∗^represents significant differences between NC, VMCI, and MCI with Bonferroni correction (*p* < 0.05/9).

We further analyzed WM-HFC to explore the specific alterations between MCI and VMCI subjects. Similar to within-group GM-HFC maps, within-group WM-HFC maps showed consistent connectivity patterns among three groups (WM-HFC_N__C_ > WM-HFC_VMCI_ > WM-HFC_MCI_) (Figure 2). Significantly abnormal WM-HFC regions were found in the bilateral middle occipital gyrus and sub-lobar and parietal lobe within WM. Specifically, compared with NC subjects, WM-HFC for the bilateral middle occipital-WM and parietal lobe-WM was lower in MCI and VMCI subjects (Figure 3 and Table 2). This result was similar to the GM-HFC results with a declining tendency. Moreover, WM-HFCs for the bilateral sub-lobar-WM were significantly lower in MCI subjects than in NC subjects but no difference between VMCI and NC.

**TABLE 2:** Regions showing abnormal homotopic functional connectivity among groups.

Brain regions	Cluster size (mm^3^)	Peak intensity (*F*-value)	MNI coordinates		
			*x*	*y*	*z*
**GM-HFC**					
Middle occipital gyrus	2,106	18.03	± 18	−90	3
Inferior parietal gyrus	918	16.97	± 30	−48	54
**WM-HFC**					
Middle occipital-WM	2,430	17.27	± 18	−90	6
Sub-lobar-WM	1,431	15.13	± 24	−21	21
Parietal lobe-WM	621	10.69	± 36	−42	33

### Anatomical Characteristics Within Abnormal Homotopic Functional Connectivity Within White Matter Regions

**FIGURE 4:**
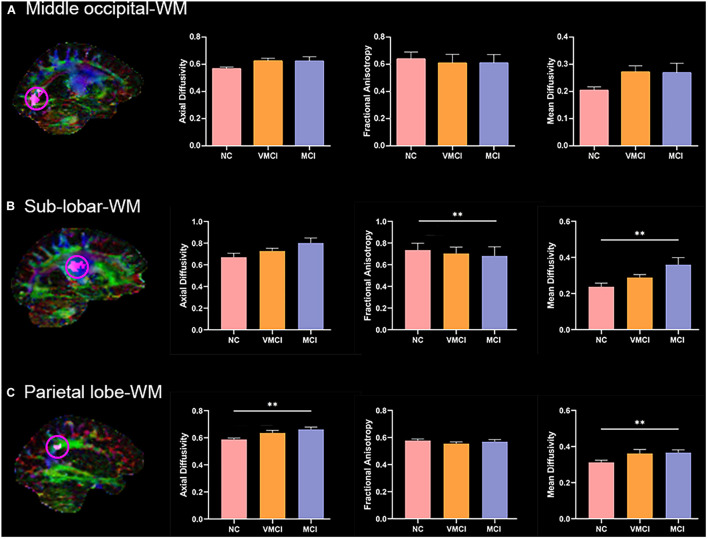
Anatomical characteristics within abnormal WM-HFC regions among groups. Magnetic resonance images maps show abnormal WM-HFC regions. Histogram shows difference of mean AD, FA, and MD within abnormal WM-HFC regions between three groups. **(A–C)** The anatomical characteristics for the middle occipital-WM, sub-lobar-WM, and parietal lobe-WM, respectively. ^∗∗^represents significant differences between NC, VMCI, and MCI with Bonferroni correction (*p* < 0.05/9).

To further explore the anatomical characteristic within abnormal WM-HFC regions, the mean AD, FA, and MD values within three abnormal WM-HFC regions were analyzed. Compared with NC subjects, MCI subjects showed a significant increase in FA and MD in the bilateral sub-lobar-WM and a significant increase in AD and MD in the bilateral parietal lobe-WM (Figure 4). Although VMCI subjects did not show significant anatomical abnormality in the WM-HFC regions discussed earlier, we found an increased tendency in the bilateral sub-lobar and parietal lobe-WM (*p* < 0.05, no-correction).

### Support Vector Machine

**FIGURE 5:**
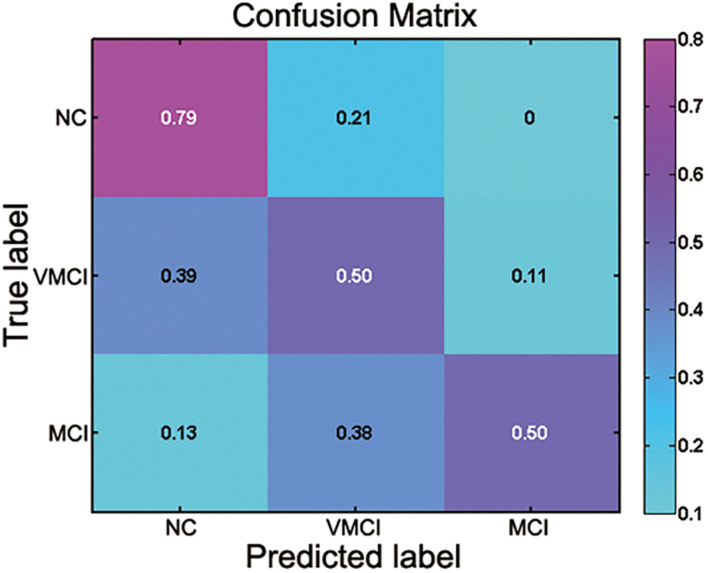
Confusion matrix for multiclass classification (NC vs. VMCI vs. MCI). Abscissa and ordinate, respectively, represent predicted label and true label.

The current study performed the multiclass SVM to identify the patient participants from NC and discriminate MCI and VMCI subjects (NC vs. VMCI vs. MCI). The accuracy of multiclass SVM was 60%. As the baseline accuracy of the three classifications was 33.33%, the 60% accuracy of multiclass SVM was moderate. The confusion matrix of results is shown in Figure 5. The NC class was the correct classifier, with only a few instances being misclassified as VMCI. The precision, recall, and F1-score values for the multiclass classification results are reported in Table 3.

**TABLE 3:** Precision, recall, and F1-score values for multiclass classification results broken down by class.

Class	Precision	Recall	F1-score
NC	57.89%	78.57%	66.67%
VMCI	60.00%	50.00%	54.55%
MCI	66.67%	50.00%	57.14%

## Discussion

In the present study, we investigated the WM-HFC in VMCI and MCI subjects and further analyzed the anatomical characteristics within these abnormal WM-HFC regions by combining the fMRI and DTI. The GM-HFC results primarily showed common neuroimaging features between VMCI and MCI subjects. Specifically, both VMCI and MCI subjects showed reduced GM-HFC for the bilateral middle occipital gyrus and inferior parietal gyrus. The WM-HFC results showed not only the common alterations but also specific changes. VMCI and MCI subjects showed reduced WM-HFC for the bilateral middle occipital and parietal lobe-WM. Specific WM-HFC abnormality was shown in the bilateral sub-lobar-WM in MCI subjects. Further, DTI analysis demonstrated that mean AD, FA, and MD within the sub-lobar and parietal lobe-WM were significantly altered in MCI subjects. Our study provided new insight into the pathophysiology of early stages of AD in relation to WM-HFC.

Resting-state HFC is a key characteristic of the intrinsic functional architecture in the human brain (Salvador et al., 2008; Stark et al., 2008). In the study, GM-HFC was estimated in the early stages of AD (Figure 1). Our results showed a decreased GM-HFC for the bilateral middle occipital gyrus and inferior parietal gyrus in VMCI and MCI subjects (Figures 1A,B). In detail, GM-HFC for NC exhibited the strongest bilateral connection, followed by VMCI subjects (GM-HFC_HC_ > GM-HFC_VMCI_ > GM-HFC_MCI_) and was consistent with previous ALFF study with ALFF_HC_ > ALFF_VMCI_ > ALFF_MCI_ (Desikan et al., 2006). One review of the available researches indicates that parietal lobe damage could cause episodic memory impairments (Cabeza et al., 2008). Moreover, visual attention deficit in AD was closely associated with decreased activation of the parietal lobe during visual search tasks (Hao et al., 2005). The abnormal GM-HFC for the bilateral middle occipital gyrus and inferior parietal gyrus might be associated with episodic memory and visual attention impairment in VMCI and MCI subjects.

Resting-state fMRI enabled us to assess temporally synchronized brain activity across local brain regions (Biswal et al., 1995; Lowe et al., 1998). Despite the prominence of resting-state HFC, few studies of WM-HFC exist. In this study, we observed decreased WM-HFC for the bilateral middle occipital and parietal lobe-WM in VMCI and MCI subjects (Figures 3A,C). We found that these two abnormal WM regions were close to the areas of GM-HFC abnormalities and showed a similar tendency in different stages of AD (WM-HFC_N__C_ > WM-HFC_VMCI_ > WM-HFC_MCI_). The abnormal WM-HFC for the bilateral middle occipital and parietal lobe-WM might also be related to episodic memory and visual attention impairment (Hao et al., 2005; Cabeza et al., 2008). In addition, the specific WM-HFC alteration for the bilateral sub-lobar-WM was found in MCI subjects (Figure 3B), suggesting that the sub-lobar-WM was crucial to distinguish VMCI and MCI subjects. Previous morphometric studies demonstrated a significant reduction of GM volume in GM sub-lobar regions in AD subjects (Ibrahim et al., 2009; Krueger et al., 2010).

To further explore the anatomic characteristics within the above abnormal WM-HFC regions, the mean AD, FA, and MD were calculated and analyzed among three groups (Figure 4). We found that the specific decreased WM-HFC for the bilateral sub-lobar-WM well corresponded to reduced FA and increased MD in MCI subjects (Figure 4B). Compared with NC, the FA values in MCI and AD subjects were significantly reduced in the sub-lobar regions extranuclear WM (Medina et al., 2006; Ibrahim et al., 2009). The sub-lobar-WM regions showed significant changes of WM-HFC and anatomic characteristics in MCI instead of VMCI, suggesting that specific regions could be used to distinguish MCI from NC. In addition, we also found that MCI subjects showed significantly abnormal AD and MD for the bilateral parietal lobe-WM (Figure 4C), which demonstrated that functional abnormality generally corresponded to structural abnormality well.

There is a growing interest in identifying the AD at an early clinical phase before dementia, with the hope that therapeutic intervention can slow the progression of neuropathological features and symptoms (Dekosky and Marek, 2003; Dickerson and Sperling, 2005; Lleo et al., 2006). The current study performed the SVM analyses to identify the MCI and VMCI subjects using the abnormal WM-HFC as the features. The accuracy of multiclass SVM was 60%. However, as the baseline accuracy of the three classifications was 33.33%, the 60% accuracy of multiclass SVM was moderate. The confusion matrix of results is shown in Figure 5. The moderate classification accuracy among NC, VMCI, and MCI subjects suggests that BOLD signals within WM can be a potential biomarker to identify brain disorders and not simply be considered as noises.

Although the findings of the present study are promising, several limitations are worth mentioning. First, several researchers have speculated that BOLD signal from WM may be infiltrated from the GM due to partial volume effect. To minimize the influence of GM BOLD signals, we performed the spatial smoothing on the WM and GM separately and used only voxels identified as WM for each subject. In addition, a strict threshold (of 0.8) was used to create a group WM mask. Second, although it was commendable for SVM analyses to use the neuroimaging data collected using another fMRI machine as a test set, the sample size for each group was too small. Future research is necessary to add enough participants to perform classification analyses. Third, as the patients in the current study were all in the early of AD (MCI and VMCI), we did not analyze the abnormal WM-HFC in AD. Finally, the current study obtained the diffusion data from 26 MCI, 44 VMCI, and 43 NC from the functional analysis dataset (53 MCI, 90 VMCI, and 100 NC), which prevented us from including the DTI parameters from the relevant ROIs as features for SVM analysis due to the lack of DTI data from all the subjects. Future study is necessary to identify the patient participants from healthy controls and discriminate MCI and VMCI subjects by combining the resting-state functional and DTI data within WM.

## Conclusion

The present study explored the WM-HFC alterations in VMCI and MCI subjects and further analyzed the anatomical characteristics within these abnormal WM-HFC regions by combining the fMRI and DTI. We found that VMCI and MCI subjects showed abnormally decreased WM-HFC for the bilateral middle occipital and parietal lobe-WM. Specific WM-HFC abnormality was shown in the bilateral sub-lobar-WM in MCI subjects. Further DTI analysis demonstrated that mean AD, FA, and MD within the sub-lobar and parietal lobe-WM showed significant alterations in MCI subjects. Our study provided new insight into the pathophysiology of early stages of AD in relation to WM-HFC. Moreover, BOLD signals within WM should not be simply considered as noises and can be a potential biomarker to identify brain disorders.

## Data Availability Statement

The original contributions presented in the study are included in the article/Supplementary Material, further inquiries can be directed to the corresponding author/s.

## Ethics Statement

Ethical review and approval was not required for the study on human participants in accordance with the local legislation and institutional requirements. The patients/participants provided their written informed consent to participate in this study.

## Author Contributions

PW and BB designed the concept and wrote the manuscript. ZW, JW, HZ, YJ, and PW analyzed the data. All authors reviewed and edited the manuscript.

## Conflict of Interest

The authors declare that the research was conducted in the absence of any commercial or financial relationships that could be construed as a potential conflict of interest.

## Publisher’s Note

All claims expressed in this article are solely those of the authors and do not necessarily represent those of their affiliated organizations, or those of the publisher, the editors and the reviewers. Any product that may be evaluated in this article, or claim that may be made by its manufacturer, is not guaranteed or endorsed by the publisher.

## Footnotes

1
https://central.xnat.org

2
http://rfmri.org/DPARSF

3
http://www.fil.ion.ucl.ac.uk/spm/software/spm12

4
http://www.fmrib.ox.ac.uk/fsl

5
http://dsi-studio.labsolver.org

6
http://rfmri.org/dpabi

7
https://www.graphpad.com/
