# Supplementary material for: Altered Homotopic Functional Connectivity Within White Matter in the Early Stages of Alzheimer’s Disease
Source: Front Neurosci. 2021 Sep 22;15:697493. doi: 10.3389/fnins.2021.697493 (PMC8492970; doi:10.3389/fnins.2021.697493)
Supplement: Supplementary file 1 [file Data_Sheet_1.docx]

**Altered homotopic functional connectivity within white matter in the early stages of Alzheimer’s disease**

Pan Wang^1^, Zedong Wang^1^, Jianlin Wang^1^,Yuan Jiang^1^, Hong Zhang^1^, Hongyi Li^2*^, Bharat B Biswal^1,3*^

1, The Clinical Hospital of Chengdu Brain Science Institute, MOE Key Laboratory for Neuroinformation, Center for Information in Medicine, School of Life Science and Technology, University of Electronic Science and Technology of China, Chengdu, China

2, The Fourth People’s Hospital of Chengdu, Chengdu, China

3, Department of Biomedical Engineering, New Jersey Institute of Technology, Newark, NJ, 07102, USA

* Corresponding author:

Address correspondence to Bharat B. Biswal, PhD., 607 Fenster Hall, University Height, Newark, NJ, 07102, USA; E-mail: [bbiswal@yahoo.com](mailto:bbiswal@yahoo.com)

Address correspondence to Hongyi Li, [M](javascript:;).[M](javascript:;). The Fourth People’s Hospital of Chengdu, Chengdu, China, E-mail: 412132904@qq.com

Running title: Altered WM-HFC in MCI

Supplementary 1

This part showed the within-group analysis results for GM-HFC.

Within-group analysis for homotopic functional connectivity within GM (GM-HFC) indicated that NC, VMCI, andMCIsubjects (Figure S1, A, B, and C) had robust regional differences (p<0.05, FDR-corrected). We found that GM-HFC maps for NC group exhibited the strongest connection, followed by VMCI group (GM-HFC_HC_>GM-HFC_VMCI_>GM-HFC_MCI_) (Figure S1), which was similar to the previous ALFF study pattern (ALFF_HC_> ALFF_VMCI_>ALFF_MCI_)(Desikan et al., 2006).


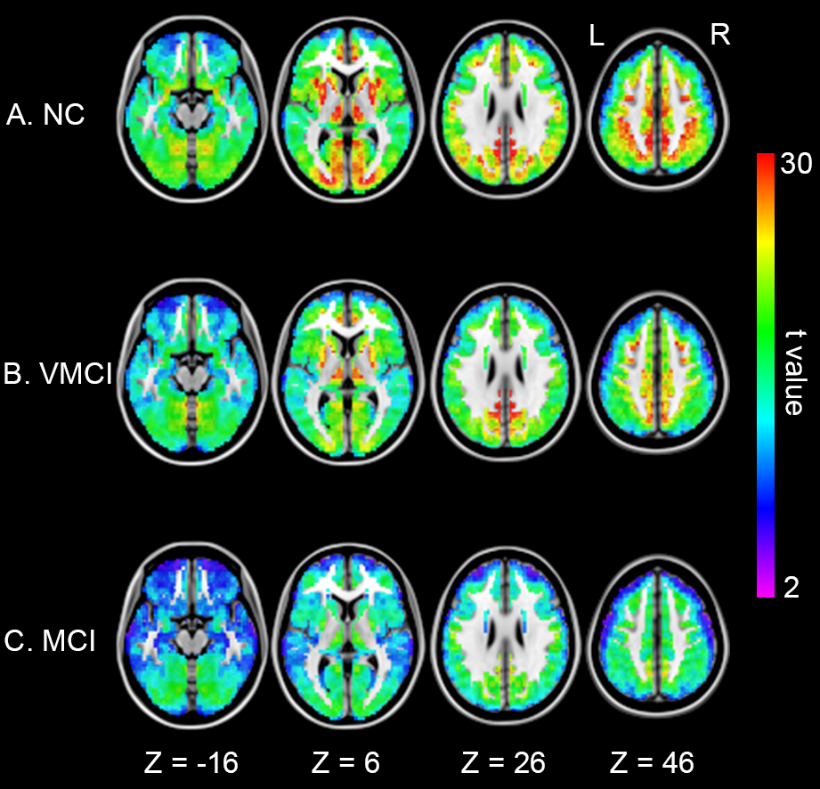


Figure S1.Homotopic functional connectivitywithin GM for each group. Colored voxels indicate that they have significant functional connectivity with the voxels at symmetric positions of the other hemisphere (p < 0.05, FDR corrected).

Supplementary 2

Thispart showed the demographics and clinical characteristics of subjects’ DTI data.

Table S1. Demographics and clinical characteristics of subjects’ DTI data

| Characteristics | CDR = 0  (N = 43) | CDR = 0.5  (N = 44) | CDR = 1  (N = 26) | P value |
| --- | --- | --- | --- | --- |
| Age | 74.16±8.232 | 76.00±7.743 | 77.88±8.071 | P = 0.2057^a^ |
| Gender (M/F) | 27/16 | 24/20 | 21/5 | P = 0.3471^b^ |
| Education | 14.12±1.905 | 14.77±2.940 | 15.12±2.861 | P = 0.0869^a^ |
| Mean FD | 0.2825±0.1504 | 0.3022±0.1756 | 0.3148±0.1489 | P = 0.2808^a^ |
| MMSE | 28.86±1.318 | 25.99±2.882 | 22.08±4.057 | P < 0.001^a^ |
| Handness (L/R) | 0/43 | 0/44 | 0/26 |  |

M = Male;

F = Female;

MMSE = Mini-Mental State Examination;

Mean FD = Mean framewise displacement;

^a^ One-way ANOVA (using nonparametric test).

^b^ Chi-square.

Supplementary 3

Thispart estimated whether excluding the subcortical nucleus from WM effected on the main results.

Since the SPM segment did not exclude the subcortical nucleus from WM, we excluded the subcortical nucleus from WM referring to previous WM functional study (Ji et al., 2019), and then further obtained the symmetric group WM mask. Based on above group WM mask, we have further estimated the effect on our results after excluding subcortical nucleus from WM. We found that the results excluding the subcortical nucleus from WM were completely consistent with our results (Figure S3).


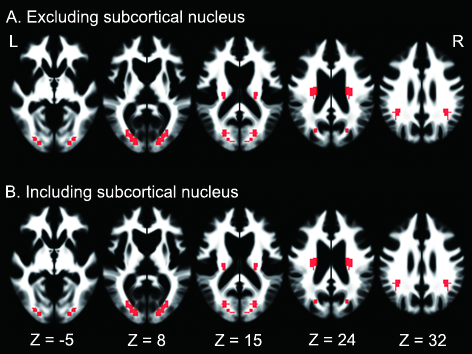


Figure S3. Comparison of results between excluded and non-excluded subcortical nucleus. A and B represent the results of non-excluded and excluded subcortical nucleus from white matter, respectively.

Supplementary 4

Thispart analyzed the correlation between the imaging and clinical symptoms, and the coupling alteration in the VMHC/anatomical characteristic.

We have analyzed the correlations between the imaging and clinical symptoms for MMSE. We found that axial diffusivity (AD) within sub-lobar-WM showed a negative correlation with MMSE in VMCI subjects (r = -0.39, p = 0.01). In addition, mean diffusivity (MD) within sub-lobar-WM also showed a negative correlation with MMSE in VMCI subjects (r = -0.38, p = 0.01) (Figure S4-1).

To estimate the coupling alteration of VMHC/anatomical characteristic in these abnormal WM regions, for each subject, we have divided the averaged VMHC by anatomical [characteristic](javascript:;) for each abnormal WM region. The VMHC/AD, VMHC/FA, and VMHC/MD in the ROIs of abnormal WM-HFC were compared between three groups using two-sample t-test (bonferroni-corrected for multiple comparisons, p < 0.05/9). Compared to NC subjects, VMCI and MCI subjects showed significantly decreased VMHC/AD and VMHC/MD ratio in the middle occipital and parietal lobe-WM (Figure S4-2, A and C). MCI subjects showed the specific decreased VMHC/FA in the sub-lobar-WM (Figure S4-2, B).


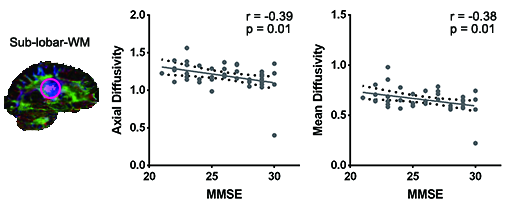


Figure S4-1. Correlations between anatomical [characteristic](javascript:;) and MMSE in VMCI. The brain map shows the abnormal WM-VMHC region for sub-lobar-WM. Two scatter plots represent the correlations between axial diffusivity/mean diffusivity and MMSE.


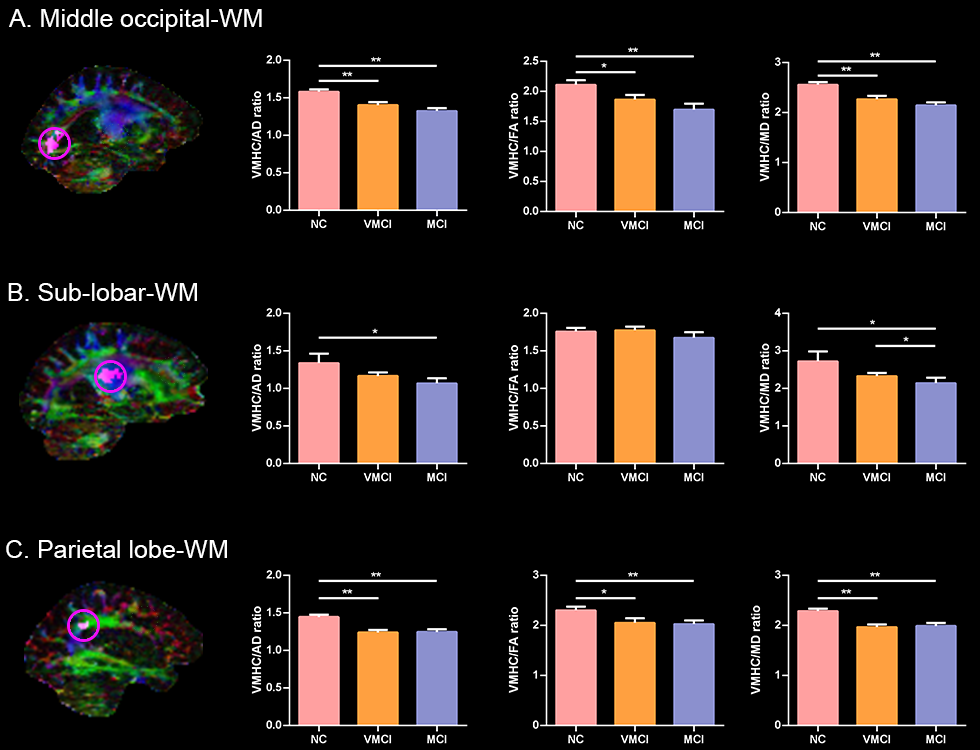


Figure S4-2. Structural and functional coupling alterations within abnormal WM-HFC regions among groups. MR images maps show abnormal WM-HFC regions. Histogram shows the difference of VMHC/AD, VMHC/FA and VMHC/MD ratio within abnormal WM-HFC regions between three groups. The statistical significance level was set at P<0.05. * and ** denote P<0.05, uncorrected and bonferroni correction (p<0.05/9), respectively.
